# Field evaluation of HIV point-of-care testing for early infant diagnosis in Cape Town, South Africa

**DOI:** 10.1371/journal.pone.0189226

**Published:** 2017-12-20

**Authors:** Lorna Dunning, Max Kroon, Nei-yuan Hsiao, Landon Myer

**Affiliations:** 1 Division of Epidemiology and Biostatistics, School of Public Health and Family Medicine, University of Cape Town, Cape Town, South Africa; 2 Centre for Infectious Diseases Epidemiology and Research, School of Public Health and Family Medicine, University of Cape Town, Cape Town, South Africa; 3 Department of Neonatal Medicine, University of Cape Town, Cape Town, South Africa; 4 Division of Medical Virology, University of Cape Town, Cape Town, South Africa; Waseda University, JAPAN

## Abstract

**Background:**

Early infant HIV diagnosis (EID) coverage and uptake remains challenging. Point-of-care (POC) testing may improve access and turn-around-times, but, while several POC technologies are in development there are few data on their implementation in the field.

**Methods:**

We conducted an implementation study of the Alere q Detect POC system for EID at two public sector health facilities in Cape Town. HIV-exposed neonates undergoing routine EID testing at a large maternity hospital and a primary care clinic received both laboratory-based HIV PCR testing per local protocols and a POC test. We analysed the performance of POC versus laboratory testing, and conducted semi-structured interviews with providers to assess acceptability and implementation issues.

**Results:**

Overall 478 specimens were taken: 311 tests were performed at the obstetric hospital (median child age, 1 days) and 167 six-week tests in primary care (median child age, 42 days). 9.0% of all tests resulted in an error with no differences by site; most errors resolved with retesting. POC was more sensitive (100%; lower 95% CI, 39.8%) and specific (100%, lower 95% CI, 98%) among older children tested in primary care compared with birth testing in hospital (90.0%, 95% CI, 55.5–99.8% and 100.0%, lower 95% CI, 98.4%, respectively). Negative predictive value was high (>99%) at both sites. In interviews, providers felt the device was simple to use and facilitated decision-making in the management of infants. However, many wanted clarity on the cause of errors on the POC device to help guide repeat testing.

**Conclusions:**

POC EID testing performs well in field implementation in health care facilities and appears highly acceptable to health care providers.

## Introduction

Every year almost 1.5 million infants are exposed to HIV. In 2015, despite global advances in prevention of mother-to-child transmission (PMTCT) of HIV programmes, almost 150,000 infants were newly infected in Sub-Saharan Africa alone [1]. Perinatally-acquired HIV infection has a high risk of rapid disease progression, and without early antiretroviral therapy (ART) more than 50% of HIV-infected infants die before their second birthday [2–4]. As a result the World Health Organization (WHO) recommends that HIV-exposed infants be tested by six weeks of life and those who test positive should immediately be referred for initiation of ART [5]. Mortality risk is especially high for those infected *in utero* [6], prior to labour and, consequently, South African National Guidelines now recommend routine testing of all HIV-exposed newborns within 48 hours of birth [7].

Diagnosis of HIV in infants is a complex process requiring multiple post-partum visits from mother/infant pairs and expensive nucleic acid amplification tests (NAAT). Inexpensive serological assays used for diagnosis in adults cannot be easily interpreted for HIV-exposed infants. The use of NAATs is strongly recommended by the WHO for diagnosis in infants, but these assays are typically performed by highly trained personnel using specialised laboratory equipment, limiting access in many high burden counties to centralized urban facilities [8–11]. Many HIV-exposed infants are therefore unable to receive an HIV diagnosis within the recommended period. In 2015 UNAIDS estimated only 51% of HIV-exposed infants received a virological test by two months of age [1]. In addition, the EID ‘cascade’ sees high levels of loss to follow-up (LTFU) at each step: offer and acceptance of testing, specimen transport and processing, result return to clinic and caregiver [8,12]. HIV-infected infants may demise before linkage to ART if the return of positive results to caregivers is delayed [13,14]. A systematic review of HIV-exposed infants traversing the EID testing cascade estimated an average LTFU of 34% but suggested that it could be as high as 75% in some sub-Saharan countries [15]. While PMTCT programmes have reduced vertical transmission dramatically, HIV-infected infants continue to have the largest gap between HIV diagnosis and treatment. A study from Cape Town found that only 71% of HIV-infected infants initiated treatment [16]. In this context, there is a clear need for novel interventions to improve retention across the EID cascade and increase the proportion of exposed infants who are tested, receive a result and, if infected, initiate ART [17,18].

The use of Point-of-Care (POC) NAAT diagnostics for EID may improve access to testing and linkage to care by reducing the turnaround time, thereby facilitating same day clinical decision making [19]. From a health systems perspective, POC EID testing has the potential to circumvent many of the logistical challenges facing laboratory-based EID systems [20,21]. However, to date most evidence on POC EID technologies comes from laboratory settings; evaluations of new diagnostic tests that take place under ideal conditions may lack real world generalizability to clinical settings across sub-Saharan Africa, and those which have taken place under field conditions fail to consider the acceptability of the device to providers [22,23]. There have been major challenges with previous attempts to implement analytical POC platforms for HIV monitoring in clinical settings, particularly when the aim is to enhance the clinical decision making of health professionals in low resource settings [24–26].

Having previously validated the Alere q in a laboratory setting [23], we performed a field evaluation to test the performance and examine the operational features and acceptability of the Alere q HIV-1/2 Detect system for EID across Cape Town, South Africa.

## Methods

We conducted a field evaluation at two public health sector health facilities: a secondary-level obstetric hospital and a primary-level midwife obstetric unit. At both sites, participants were HIV-exposed children under 1 year of age. At the time of the study, infant HIV PCR testing was performed routinely in (i) all HIV-exposed infants at 6 weeks of age and (ii) at birth for HIV-exposed infants classified as at high risk of vertical transmission per local guidelines [27]. Consecutive infants were selected for HIV testing on both laboratory based assays and POC assays in parallel. Signed informed consent was obtained from a parent or guardian for their child to participate in the study.

We compared the Alere q 1/2 Detect (Alere Technologies GmbH, Jena, Germany) POC device to the local standard-of-care (SOC) for EID testing, the Roche Cobas AmpliPrep /Cobas TaqMan (CAP/CTM) HIV-1 qualitative assay (Roche diagnostics, Branchburg, New Jersey, USA). POC tests were completed at either a large primary-level community health centre or a secondary-level obstetric hospital with neonatal care facilities. SOC was conducted at the National Health Laboratory Service virology laboratory at Groote Schuur Hospital, Cape Town.

The Alere q HIV-1/2 POC test is comprised of a disposable cartridge and analyser platform (“Alere q”) [28,29]. Whole blood samples were collected in EDTA microtainers via venepuncture from infants undergoing routine HIV testing per local protocols. 25μl was removed via a capillary tube and deposited into the disposable POC cartridge prior to transport to the central laboratory for SOC testing. No additional samples were drawn from infants for participation in the study. During the study period the manufacturer provided technical support for any breakdown in the device, and provided the initial training to providers on how to operate the platform, thereafter these first users trained new staff who joined during the study period.

As part of the evaluation, eight providers who had used the machines regularly were interviewed using semi-structured interviews to collect qualitative data on the acceptability of the device to users. Each interview was conducted in a private space using an interview guide and lasted approximately 30 minutes (S1 Text).

The study was approved by the Human Research Ethics Committee of University of Cape Town Faculty of Health Sciences and the Provincial Government of the Western Cape. The manufacturer loaned machines and donated cartridges for this evaluation, but provided no other material support and played no role in study design, conduct, analysis or reporting.

Data were analysed using Stata Version 12.0 (Stata Corporation, College Station, Texas, USA). Some infants provided multiple specimens and underwent multiple point-of-care tests, in analysis, we included the results of the first point-of-care result for each specimen directly compared to the standard of care result from the laboratory, we subsequently included additional POC test results if the original resulted in an error message. We excluded any specimen associated with an indeterminate SOC result from the analysis, as they necessitated further tests with additional samples. We also excluded any samples run on a faulty platform from the overall analysis but consider these results separately. All faulty devices were replaced by the manufacturer and an alternative device used for the remaining study period. An infant was considered HIV-infected if a sample tested positive on two different NAAT assays or a subsequent specimen conclusively tested positive on the SOC assay. Test performance for all infants, was stratified by testing facility, and estimated using sensitivity and specificity as well as positive and negative predictive values, with exact 95% confidence intervals (CI). Operational features including average daily demand, average number of tests per patient and error rates were estimated as proportion of total number of samples or patients tested. Throughout, Fisher’s exact tests were used to compare proportions; all statistical tests are 2-sided at alpha = 0.05. Qualitative data were transcribed from interview recordings, with audio records cross-checked to ensure validity. Researchers assigned specific codes to the transcript text to bookmark key concepts discussed in the interviews; representative quotes are provided to demonstrate key themes from interviews.

## Results

In total, 478 specimens from 453 children were tested on the POC platform between December 2013 and August 2014 at the two facilities. One Alere q machine was used at the obstetric hospital and two further machines were used in parallel at the primary care community health centre. In both settings, the device was located in side rooms adjacent to patient consultation rooms.

**Fig 1:**
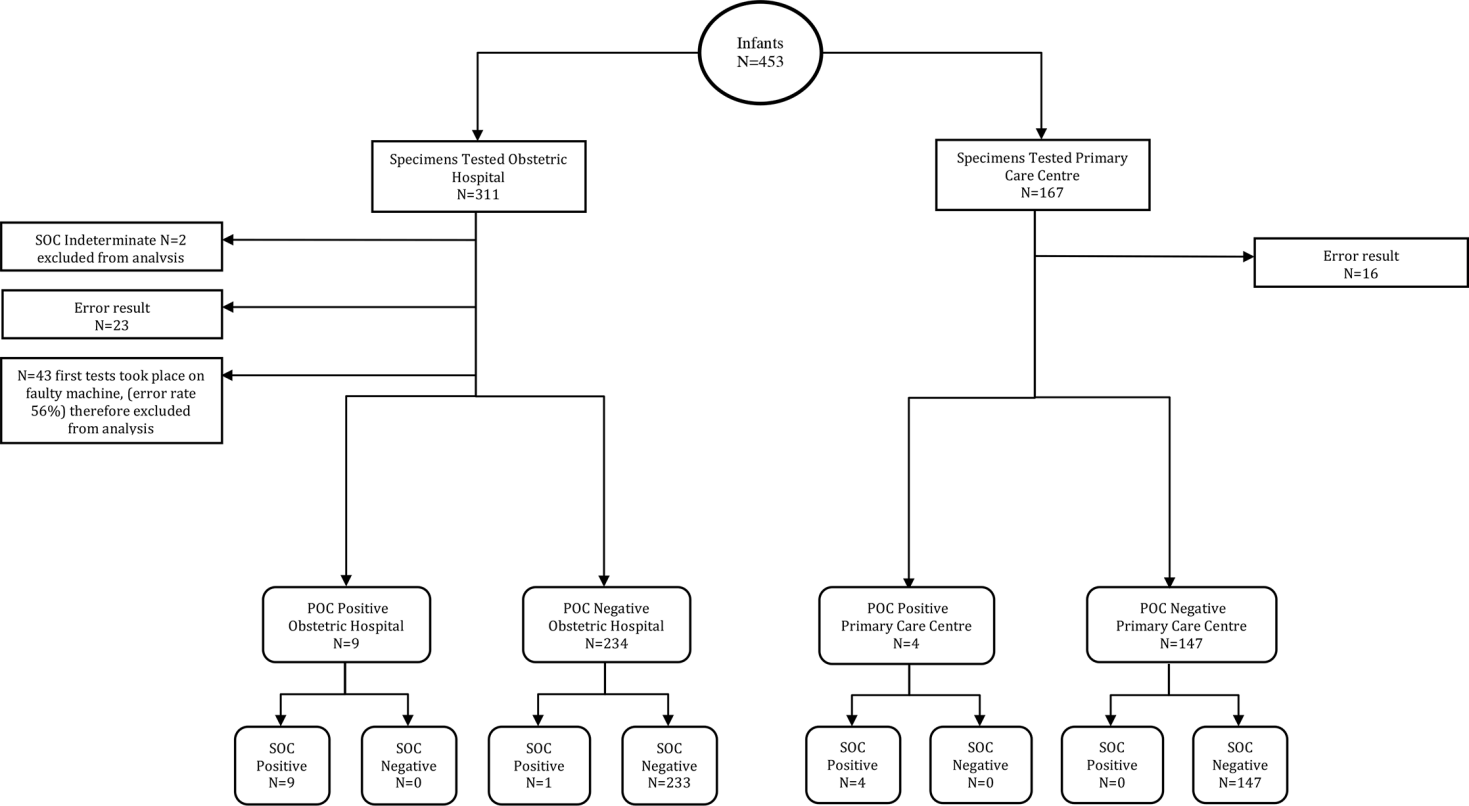
STARD diagnostic flow diagram. Infants receiving an HIV PCR test as per local protocol were tested on the POC device and at the laboratory. Some infants provided multiple specimens and underwent multiple point-of-care tests. This diagram reflects the first point-of-care result for each specimen which is directly compared to the standard of care result from the laboratory. Tests are stratified by field testing site.

The diagnostic flow diagram for the 453 infants tested using POC is shown in Fig 1. Two SOC results were recorded as indeterminate. Additionally, 43 infant specimens run on a faulty instrument (increased error rate of 55.8%; n = 24), used initially at the obstetric hospital between 9^th^ December 2013 and 8^th^ February 2014, were excluded from the main analysis.

Of the infants who contributed specimens for the 266 first tests at the obstetric hospital the median age was 1 day with 96% (n = 256) of specimens taken from newborns (age <7 days).

In comparison at the primary care facility, the median age was 42 days, with only 11% (n = 19) of specimens taken from newborns (age <7 days) and 54% (n = 90) of specimens tested as part of routine EID (ages 6–14 weeks) (Table 1). HIV-exposed infants provided 433 first specimens to be tested on the POC platform, 13 returned a positive result, 39 resulted in errors and 381 recorded a negative result on the first test giving overall positivity rate of 3.0%. At the primary care community health centre 2.4% (4/167) of specimens were positive by SOC and POC, whilst at the obstetric hospital 4.9% (13/266) were positive on the SOC but only 3.4% (n = 9) of POC first tests returned a positive result, 1 sample returned a false negative result, and 3 infants returned error results but were identified as infected on subsequent POC tests (Table 2).

**Table 1:** Summary study population demographics stratified by health care facility. Infants received a routine EID test (SOC) at either the obstetric hospital or primary care centre. The specimen used for routine testing was also used for POC testing in parallel within the field setting (SOC: Standard of Care).

		Obstetric Hospital N = 266 (%)	Primary Care Centre N = 167 (%)
SOC result	Positive	13 (4.9%)	4 (2.4%)
	Negative	253 (95.1%)	163 (97.6%)
Age group	<7 days	256 (96.2%)	19 (11.4%)
	7 days to 6 weeks	6 (2.3%)	42 (25.1%)
	6–14 weeks	4 (1.5%)	90 (53.9%)
	>14 weeks	0 (0%)	16 (9.6%)
Sex	Male	127 (47.7%)	76 (45.5%)
	Female	115 (43.2%)	88 (52.7%)

**Table 2:** Performance of Alere q Detect HIV-1 (first test) against the standard of care Roche CAP/CTM HIV-1 PCR.

Obstetric Hospital				Primary Care Centre			
Alere q	Roche CAP/CTM HIV-1 PCR			Alere q	Roche CAP/CTM HIV-1 PCR		
	Positive	Negative	Total		Positive	Negative	Total
Positive	9	0	9	Positive	4	0	4
Negative	1	233	234	Negative	0	147	147
Error	3	20	23	Error	0	16	16
Total	13	253	266	Total	4	163	167

### Operational characteristics of POC device

The average daily POC test demand at the two sites was 2.0 per day at the obstetric hospital and primary care facility. Maximum daily demand at both sites was shown to be 7. Tests were performed by 29 users at the obstetric hospital (many of these were transient medical officers who performed very few tests each). All users at the obstetric hospital were physicians, whilst the 3 users at the primary care facility were a senior research nurse and two healthcare assistants (Table 3).

**Table 3:** Operational features of Alere q Detect HIV-1 performed at the obstetric hospital and primary care centre. Numbers reflect total tests run, including repeat tests for specimens recording an error message on the first attempt, but exclude samples run on faulty device.

	Obstetric Hospital	Primary Care Centre
**Number of Tests**	285 (266 first tests)	185 (167 first tests)
**Number of Breakdowns**	1	0
**Average Daily Demand**	2.02	1.95
**Peak Daily Demand**	7	7
**Number of Users**	29	3
**% of tests HIV+**	3.4%	2.4%
**Sensitivity**	90% [95% CI, 55.5–99.8%]	100% [95% CI, 39.8–100%]
**Specificity**	100% [95% CI, 98.4–100%]	100% [95% CI, 97.5–100%]
**Positive Predictive Value**	100% [95% CI, 66.4–100%]	100% [95% CI, 39.8–100%]
**Negative Predictive Value**	99.57% [95% CI, 97.7–100.0%]	100% [95% CI 97.5–100%]
**Error Rate**	0.086	0.096

At the primary care facility, the sensitivity and specificity the of Alere q were both 100% (lower 95% CI 39.8%, & lower 95% CI 97.5% respectively). At the obstetric hospital, which, due to risk-based birth testing, has a considerably higher prevalence of infant HIV infection, the sensitivity of Alere q was 90.0% (95% CI 55.5–99.8%) due to one false negative result, but specificity was found to be 100% (lower 95% CI 98.4%). If final test results from multiple POC tests after errors were included in the sensitivity calculation at the obstetric hospital rather than only the first test the sensitivity increased to 92.3% (95% CI 64.0–99.8%). For infants who received a result the POC assay was 99.6% accurate at the obstetric hospital and 100% at the primary care facility.

An error message was generated on the first attempt for 9.6% (n = 16) of infants at the primary care facility and 8.6% (n = 23) of infants at the obstetric hospital (Tables 2 & 3). Not all tests were able to be repeated due to inadequate sample volume, but of those which were able to be retested 13/16 (81.3%) were resolved after one additional test at the primary care facility and 2/2 (100%) errors were resolved after two additional test. Only one child was unable to receive a POC result from the Alere q at the primary care facility. At the obstetric hospital 10/16 (62.5%) were resolved after one additional test, and 2/3 (66.6%) were resolved after two additional tests, meaning 11 infants never received a POC result.

Results from a faulty device initially placed at the obstetric hospital had a very high error rate, and were excluded from the overall analysis. They included three positive (7.0%), 16 negative (37.2%) and 24 (55.8%) error results. No discordant results were found, but 18 infants would not have received a POC result within a three-month period when this faulty prototype device was being used.

### Provider acceptability

Three main themes emerged during qualitative analysis: feasibility, practicality, and empowerment. All providers said the technology was very easy or easy to use, all mentioning the limited training required to be able to use the device. 75% of interviewees spoke about the advantages of only requiring a small samples size and 88% mentioned the benefits of being able to print out the results slip.

“*The machine does it all*, *it was very simple*, *I mean if you can use a cell phone then you can use this*”

“*The print out was helpful and could be placed in the patient booklets*”

“*It was nice that only a small amount of specimen was required*”

However, practically many of the providers were frustrated by some of the design features, 75% of interviewees said the ejection mechanism of the cartridge had caused problems and 25% said the design of the keyboard was irksome. All providers interviewed said the lack of information given when specimens aborted the cycle and recorded an error message was frustrating.

“*There were some problems with the key board and the ejection of the cartridge*”

“*It was hard to know if the machine wasn’t working or if it was a user problem*”.

The quantitative data analysis found high error rates of around 9%. However, this did not seem to be a problem with the providers. 88% of providers interviewed said that the number of errors was acceptable and had not impacted their experience using the POC device. 50% said they felt there was a “*knack*” to running a cartridge correctly, and if the specimen aborted the perception was that running a new sample immediately afterwards would return a result.

“*With the error codes we wouldn’t know what the issue was, but if we then ran the specimen again, I mean I personally can’t think of any that I had that didn’t work the second time*”

All providers said they would use the Alere Q for EID if it was available. One interviewee from the obstetric hospital spoke about the impact on LTFU if POC was used rather than conventional laboratory techniques;

“*We don’t want to send mothers/infants away without a result so for the staff and for me it would be good to be able to get a result and know what treatment was needed for the baby.*”

A provider from the primary care clinic spoke about a sense of relief for themselves at having an instant result.

“*It would be nice to not have to worry about the children who are at risk, if I get a result straight away… means that I don’t have to worry so much*”

## Discussion

These data reflect a growing field of implementation of POC devices in field settings. There were three key findings. First, HIV diagnosis can be accurately carried out in a range of public healthcare facilities by non-laboratory personnel. Point-of-care tests demonstrated high sensitivity and specificity both in newborns and routine EID. Second, health care professionals found the platform to be feasible and practical to use in a clinical setting. However, we found error rates of the Alere q POC platform were higher across the two field settings than seen in previous validation studies, raising concerns around cost and quality control.

Our data demonstrated the POC NAAT platform to have perfect specificity and high sensitivity in both hospital and primary clinical care, indicating that it can be used by a variety of health care professionals, without variation in results. As PMTCT programmes widen their coverage across sub-Saharan Africa the accessibility and decentralisation of testing facilities is increasingly important. Capacity to perform HIV NAAT tests accurately at point-of-care using non-laboratory personnel in field settings decentralizes services and reduces the steps involved in the cascade, aiding prompt initiation of ART for infected infants and limiting LTFU. This is particularly pertinent in birthing facilities where congenitally HIV-infected newborns may be identified and commenced on treatment before discharge home.

Perfect sensitivity of the POC platform was found at the primary care community health clinic, and a slightly lower sensitivity (90.0%) was calculated from the obstetric hospital. These data points were affected by the low numbers of HIV-infected infants and therefore have wide confidence intervals but are comparable to laboratory validations [23]. Tests performed at the primary care facility were mostly routine tests at six weeks of age whereas most tests at the obstetric hospital were risk-based tests performed within 48 hours of delivery, explaining the higher yield of positive results. We were unable to ascertain the reasons for the false negative result at the obstetric hospital, but low viral copy numbers or mutations of the virus located in the primer sequence have previously been found to prevent the correct identification of infected infants [30,31].

The POC platforms returned no false positive results. High specificity is critical to using a diagnostic test for HIV diagnosis in infants. False positive results are becoming an increasing concern in settings with low MTCT rates resulting in lower positive predictive values of diagnostic assays [32,33]. Infants incorrectly diagnosed as infected who initiate lifelong ART have limited opportunities to be correctly identified as truly uninfected once ART initiation has occurred. This can place a considerable burden on both infant, their family and the wider health system [33]. The WHO strongly recommends prompt ART initiation after a first positive result but that a NAAT test be repeated on a second specimen, drawn prior to ART initiation, to confirm the result. POC testing could allow infected infants to initiate ART without delay at the same clinic visit (regardless of whether a SOC or POC test is required for confirmation) during a period where delay incurs high mortality. However, balancing retention in care of infected infants with the costs of conventional and POC testing will be critical for policy makers and programme managers.

Although the overall performance of the test was equivalent to the laboratory validation, the proportion of results giving an error message is higher in the field setting (9.0% vs. 6.0%), and considerably higher than the accepted error rate on SOC platforms of 2–3% [23]. The device used was a prototype, and most errors were resolved upon retesting. No operator was associated with higher error rates indicating no systematic cause. It is possible the higher error rate may be linked with sample preparation in the field prior to placement in the cartridge. The laboratory study included laboratory trained personnel using pipettes to deposit the sample into the disposable cartridge whereas this field study used plain capillary tubes. Although the higher number of errors recorded in this study raises concerns of cost and quality control, it did not seem to affect the acceptability of the device. In general, providers were extremely accepting of the Alere q platform. They found it simple to use and managed the extra workload of running the platform along with their other duties. Many felt using the POC result enhanced efficiency and would have enabled them to make quicker and more effective decisions on patient management. However, all providers interviewed expressed frustration around the lack of feedback information provided when an error occurred. Better understanding of reasons for errors and ways to correct specimen collection or operator associated errors will be important as POC testing is implemented in more remote settings.

The study highlights important operational considerations of cost effectiveness and quality assurance systems [26]. The possibility of incorrect results and high errors rates would require a stringent quality assurance system which should be included in any cost calculations before POC could be named as the preferred method for EID in South Africa. Moving forward POC implementation requires a careful balancing act and POC EID technology needs to be used in conjunction with laboratory-based testing [25]. POC testing may have particular application in remote settings and in birth facilities where every HIV-exposed neonate can be discharged with a first result and infected infants can be referred or retained for immediate ART initiation.

There are several limitations to the study. First, the use of a prototype device in this study means the performance characteristics may be underestimated compared to the regulatory approved device. The use of a prototype also meant we were unable to return POC test results to mother/infants pairs during the validation process and thus were unable to ascertain the impact of the EID POC device on infant outcomes. Second, the small sample size and low transmission rate meant very few positive samples were tested on the device during the study period. Third, we did not have access to information on maternal ART use, nor on antiretroviral prophylaxis given to infants, and thus cannot comment on the potential role of antiretrovirals in influencing both POC and SOC EID test results. Finally, our study did not include comparison with SOC assay performed on DBS which is widely used in low resource settings. As both DBS and POC use whole blood samples rather than plasma as in SOC, DBS results are likely to compare more favourably with our results.

In summary, this study suggests that the Alere q HIV1/2 Detect prototype performed well in field settings in a challenging population previously found to have high coverage of maternal ART and infant prophylaxis [34]. The POC device was not only acceptable to providers but even favoured over routine SOC. The Alere Q was found to have high sensitivity and specificity for both routine 6 week EID and birth testing. The small number of HIV-infected infants highlights the progress being made towards the elimination of mother to child transmission but is a concern for all EID validations. Emphasis should be placed on the importance of collaborations such as the EID consortium to expand the number of positive samples included in validation of POC devices and avoiding delays in the use of POC in field settings. Future research should focus on implementation and operational aspects of this important technological innovation, particularly in settings remote from central laboratories and birth facilities.

## Supporting information

S1 Data Data file. (XLSX)

S1 Text Interview guide for the evaluation of HIV point-of-care testing for early infant diagnosis in Cape Town, South Africa. (PDF)
