# Supplementary material for: Field evaluation of HIV point-of-care testing for early infant diagnosis in Cape Town, South Africa
Source: PLoS One. 2017 Dec 20;12(12):e0189226. doi: 10.1371/journal.pone.0189226 (PMC5738050; doi:10.1371/journal.pone.0189226)
Supplement: S1 Text — (PDF) [file pone.0189226.s002.pdf]

Field evaluation of HIV point-of-care testing for early infant diagnosis in Cape Town, South Africa

Interview Guide

Script

Thank you for your participating in the qualitative arm of our study today. This interview will take up to 30 minutes and will include 9 questions regarding your experiences of working with the point-of-care device. If at any time you need to stop or take a break, please let me know. There will be no consequences for withdrawing your participation.

I would like to ask your permission to record this interview, so I may accurately document the information you convey. All of your responses are confidential and will be used to develop a better understanding of the acceptability of the device to providers.
If at any time during the interview you wish to discontinue the use of the recorder, please let me know.

I would like to confirm that you consent to participating in this part of the study and your participation in this interview is completely voluntary.

Do you have any questions or concerns before we begin?

*****

Interview #_______________
Date_______/_____/_______

Question 1.
Over what period did you use the point-of-care device in your work?

Question 2
How did you feel about being asked to use the POC device?

Question 3
How did this differ from your normal tasks? Probe for workload as HIGHER, LOWER or SAME as before the study started?

Question 4.
How long did it take for you to feel confident to use the POC device?
Question 5.
How would you rate your overall experience using the device?

Question 6.
Could you explain why you rated your experience as ____ ?

Question 7.
Thinking about your previous answers, what factors would you specifically identify as influencing your experience? (probe for deeper answers if question not satisfactorily answered… Please explain why you think these are important factors? Positive or negative influences? Do you think the design of the device is influencing? The time taken to get a result is influencing?)

Question 8.
Would you continue to use the device if it was available within this facility?

Question 9.
Before we conclude this interview, is there anything else you would like to share about your experience using the point-of-care device?

Thank the participant for his/her participation.
